# Magic Money’s Theater of Time: Games, Fiction, and the Ethics of Digital Economies

**DOI:** 10.1007/s12115-026-01196-y

**Published:** 2026-04-30

**Authors:** Dorothea Rebecca Schönsee

**Affiliations:** University of Vienna, Vienna, Austria

**Keywords:** Speculative economies, Digital assets, Thomas Pynchon, Neal Stephenson, Gamic fiction, Cryptocurrencies

## Abstract

Contemporary fiction has not only inspired early experiments in cryptography and digital finance but has also exposed the intentional conflation of mystery and conspiracy for the sake of new forms of extraction of time from the nexus of games, finance, and digital assets. Drawing on Boltanski and Chiapello’s *The New Spirit of Capitalism* and Boltanski’s investigation into *Mysteries and Conspiracies*, this paper reads Neal Stephenson’s *Reamde* (2011) and Thomas Pynchon’s *Bleeding Edge* (2013) as critical tests of the reconfigured spirit of capitalism in digital economies. This hybrid regime justifies value through narratives of magical freedom, emotional intensity, and player agency while obscuring parasitic control, exploitation, and neo-colonial extraction. Stephenson constructs this justification through libertarian hacker immersion and reputational capital, framing disruption as justificatory. Pynchon exposes that myth as a vehicle for crime, terror, and predation: Hackers warn of surveillance and its fateful implications, but they ultimately fail to solve the problem. Gaming spaces slowly overtake reality as a hopeful but illusory way to rebuild trust, revealing divergent escapes—into either a domesticated nature or the memory archives of the past as projective maps of a digital future. By comparison, the texts reveal how the emotional turn in digital value erases historical and collective memory while sustaining the illusions of digital regimes. Their critical tests expose a mystery economy in a simulated theater of time interrogating how the ethics of digital economies shape the future of social trust depending on the availability of opacity and privacy.

## Introduction: Virtual Assets in Gaming Worlds of Fiction

In digital economies, the themes of mystery and conspiracy link to inquiries of circumvention and distrust of “real-world” governance, mobilizing instead worlds of virtual magic and ludic powers. Whether earned in gaming economies or generated in algorithmic systems, digital assets become entities that store value and increasingly structure the apprehension of time in mediated realities. They appear superficially as guardians of new magic money and as revolutionary, disruptive actors in economic networks. Nevertheless, they are often revealed to carry deceptive schemes or are themselves prone to fraud and hacks, such as the rug-pull scams or ransomware attacks rattling crypto markets. This ontological tension becomes in itself the setup for a theater of time.

While the use of different types of performativity by digital economies has received substantial attention (Lee, 2022; Unal et al., 2025), the analytic value of literary fiction functioning as a critical test of how digital economies capitalize on conspiracy and mystery remains largely unexplored. Likewise, the role fiction plays in prefiguring or shaping remailers and early concepts on digital cash has received only limited scholarly attention (Brunton, 2019, pp. 79–83). This is surprising given that hackers and cypherpunks frequently cite fiction as sources of inspiration for their experiments on pseudonymity and remailers.

Thomas Pynchon and Neal Stephenson are frequently mentioned in discussions about encrypted anonymity and decentralized networks (May, 2001, p. 38). Not until 2003 did cypherpunk Timothy May voice doubts, asking, “Pynchon as roadkill on the digital highway?” (May, 2003). At that time, it dawned on him that the movement’s embrace of the notoriously private writer—“Pynchon, not photographed since his high school yearbook photo 40 years ago, a total cipher” (May, 2003)—could backfire via exposure in a reported data leak of Pynchon’s purported address, ironically strengthening the justificatory regime of capitalism. On the same thread, Cyrus Shaoul (2003) cautioned against trusting the data, highlighting the core issue, namely that data remain open to interpretation regardless of any leak. Others praised Neal Stephenson’s privacy-enhancing techniques. In alignment with these reflections, fiction shifted from justifying digital cash to critically testing digital economies.

Drawing on Luc Boltanski and Ève Chiapello’s *The New Spirit of Capitalism* (1999/2007) and Boltanski’s *Mysteries and Conspiracies* (2012/2014), an investigation of detective fiction, this article aims to address this research gap by analyzing Stephenson’s *Reamde* (2011) and Pynchon’s *Bleeding Edge* (2013/2014) as exemplary, yet complementary, critical tests of the reconfiguring spirit of capitalism in emerging digital economies. For Boltanski and Chiapello, the *New Spirit of Capitalism* is characterized by the inclusion of critical attitudes and neo-avant-garde aesthetics into new inquiries and tests. These attitudes solidify the very capitalism they had set out to disrupt—that is, a spirit previously alien to the system is integrated into a justificatory regime, or an ideological tool. “The spirit of capitalism is precisely the set of beliefs associated with the capitalist order that helps to justify this order and, by legitimating them, to sustain the forms of action and predispositions compatible with it” (Boltanski & Chiapello, 1999/2007, p. 10). This justificatory function finds a new expression in fictional realities: The theatrical narration of mystery functions as a set of justifying beliefs to a new spirit brought about by cryptography and a shifting perception of society becoming posthuman (Hayles, 1999/2002).

My main thesis is that the aesthetic critique given by Stephenson and Pynchon, who are both influential to the crypto imaginary, exposes how digital economies replace historical memory with artificial mythologies: as a deliberately staged mystery in a theater of time built on paranoia and conspiracy. These conspiracies legitimize an emerging structure, which eventually evades privacy and questions opacity. Contemporary *market metafictions* (Crosthwaite, 2019, pp. 35–68) and gamic fictions (Jupin, 2014; Stark, 2019) then expose how digital economies present themselves as mystery economies for a new gold rush, betting on a blending of the secret with the token, on the cryptographic riddle, to legitimize their playing field, prefiguring observations of centralization within purportedly decentralized finance (Langley & Leyshon, 2021, p. 380). The conflation of mystery and conspiracy in fiction extends the test of justification for fragile realities beyond diagnostic exposure by incorporating artistic dissonance to reconfigure ontological boundaries and to deepen sociological critique.

In both texts, digital assets function as supervised tests for an emotional turn and temporal attachment. In Stephenson’s *Reamde*, the MMORPG game T’Rain, modeled after *World of Warcraft* (Stephenson, 2011, p. 33), serves as a test of extraction regimes, drawing outside and inside conspiracies and terrorist actors into one unified gaming network. In *Bleeding Edge*, the detective story set against the ongoing effects of the dot-com bubble burst and the events of 9/11 unravels how much the spirit of capitalism devours spaces of imagination under the guise of conspiracy. Given that digital assets increasingly partake in a contemporary theater of time—performing progression, stalling, and change—and become narrative actors, it becomes imperative for literary critique to ask how their performances of value reconfigure our view on cultural histories they potentially lead away from in creating history-free realities, raising the issue of which parts of the social structure they displace or even “overwrite.”

The staged mysteries of digital money raise additional questions about the role encrypted privacy and opacity play within the aesthetic tests of these novels as a politicized strategy of resistance against “reductive transparency” (Glissant, 1990/1997, p. 62) as the new spirit of digital capitalism. As both novels portray an exit to capitalist regimes—Stephenson into the no-man’s land and domestic world, and Pynchon into an enigmatic core—the issue that derives from the perspective of criticism is how and for whom an exit from the projective regimes in both scenarios is even possible. Between the performative capitalist exits and the aim of protecting privacy, exit and opacity emerge not only as an aesthetic choice but also as an ethical intervention on who is given a voice and whose voice and body are muted. Given that opacity for some may be a condition of survival, the comparison thus aims to sharpen a question that may be buried in Pynchon’s detective enigma and demarcated by Stephenson’s thriller: What is the difference between choosing to go dark and being denied the option of visibility and voice?

## Theoretical Framework: Literary Sociologies and the Test of Mystery Economies

Following Boltanski’s *Mysteries and Conspiracies*, both *Reamde* and *Bleeding Edge* occupy a space between detective fiction, a diagnostic genre, which employs mystery “detached from the background of a stabilized and predictable reality whose fragility is revealed by crimes” (Boltanski, 2012/2014, p. xv) and the obfuscation of the normative realities emerging from conspiracies by introducing picaresque elements. These picaresque snippets twist the discovery so that it is always already a fool’s game, subverting all actors to a game of chance rather than logic (Boltanski, 2012/2014, pp. 7–8). This hybrid positioning allows the novels to diagnose and equally disrupt the justificatory logic of capitalism, while portraying the “difficulty in establishing a scale of justice in networks as it is not always known who is on the inside and who is on the outside” (Boltanski & Chiapello, 1999/2007, p. xiii). Digital money, crypto-schemes, and video games emerge as a new form of supervision in which critique transmutes into emotional intensity, turning the supervised test of writing into one of temporal detachment. In these novels, games and conspiratorial narratives perform this test of strength, in which detachment of time turns into a mode of affluence and accumulation. Thus, the portrayal of digital economies aligns with what Boltanski and Chiapello (1999/2007, p. 510) describe as destructive effects in the “unfettered resumption of accumulation,” asserting that “released from control, without constraints, capitalism knows no criteria except the private interests of the strongest and has not reason to take account of the general interest.” They illustrate that “critique thus plays an important role in the formation of the spirit of capitalism, which, if it is to be credible, must have corresponding supervised tests” (p. 490). Hence, these novels center on the core problem of the *New Spirit of Capitalism*: the co-option of critique by means of emotional mystery. This conceals the private interests of the strongest, which are now legitimized through different conspiracy theories, as portrayed most pronounced in *Bleeding Edge*. While Stephenson (2011, p. 34) frames this through libertarian gaming utopias providing a “currency exchange scheme” but disrupted by hackers, Pynchon portrays conspiratorial money laundering associated with cryptocurrencies and the dark web (e.g., *Silk Road*). Computer scientists and hackers were fascinated with such visions, as evident when the technical writer Robert Hettinga (1996/2003) praised Stephenson for his ability “to create utterly […] novel, reality out of whole cloth” and described “in perfect detail, a geodesic economy—complete with digital cash, payer anonymity, and money as software food,” a topic which re-emerges in *Reamde.* Both writers expose how mystery economies legitimize the strongest actors while displacing historical memory through pseudo-spiritual readings while using disparate writing strategies: Stephenson’s novel builds on a narrative of an exit strategy. Thus, protagonists acquire reputational or “avatarial capital” (Nakamura, 2009, p. 136) through pseudonyms in games. However, the players’ respective exits also constitute the rules of the new spirit of the digital economies portrayed.

In contrast to *Reamde*, *Bleeding Edge* functions as a test of exposure: Its hacker utopia, DeepArcher, is already co-opted. Pynchon’s detective heroine and fraud investigator, Maxine Tarnow, finds no clean resolution and conspiracy dissipates into mystery and amnesia. On the other hand, Stephenson’s protagonists retreat into an Emersonian dream of nature as language beyond cryptography, establishing trust through essentialism. Stephenson’s ransomware thriller *Reamde* and Pynchon’s *Bleeding Edge* expose how the critic’s task is not so much unravelling the mystery as recognizing that the mystery offers a frame in its own right, one that never fully reveals its crime and thus preserves that fragility. Pynchon, quoting from Donald E. Westlake suggests in the epigraph to *Bleeding Edge*: “New York as a character in a mystery would not be the detective, would not be the murderer. It would be the enigmatic suspect who knows the real story but isn’t going to tell it” (Pynchon, 2013/2014). Its *metareality of money* (Pöhlmann, 2016) becomes equally suspect, creating its own story and reality in a liminal space that may be equated with what Boltanski and Chiapello call a *projective city*. The projective city, DeepArcher, is a space of contradictions and antinomies and offers freedom only to those who can surrender their past as an unresolvable antinomy at the heart of digital sanctuary dreams.

Despite these divergent outcomes, both novels engage the same underlying tension between social critique and co-opted aesthetics. Both texts revolve around the emerging digital economies of virtual space and digitalization by homing in on the convergence of artistic critique, autonomy, creativity, authenticity, and liberation. They are recuperated by capitalism, which simultaneously blocks any “social critique” (Boltanski & Chiapello, 1999/2007, pp. 326–327). Both texts explore how digital economies thrive in destructive expansion (p. 531), transforming game-based spaces into projective virtual cities. In doing so, they highlight the key difference between performative temporality in a projective terrain and fiction’s inert opposition to it as a slow medium. Despite their different artistic compositions and genres, both novels perform temporal modulations that become spatial critique. They demonstrate that once digital assets convert time into stories of magic and emotion, aesthetic critique becomes an important tool. It differentiates between projective cities, as they subsume agency under schemes of terror and strength, and between the aesthetic utopias of preserving a spirit of the collective commons, to remain within an open domain, rather than forcing a flexible workforce into *chokepoints of capitalism* (Doctorow & Giblin, 2022).

The emerging digitalized financial regime implements aesthetic strategies to obscure time regimes, framed as a justificatory hybrid in digital capitalism. This temporal obfuscation becomes evident in both texts. Stephenson (2011) portrays how value is derived from staged paranoia and conspiratorial narratives, that is, the hide-out from criminal prosecution in no-man’s land. Pynchon (2013/2014, pp. 192–193, 242, 431) deploys the Montauk conspiracy theory as a backstory to his ambivalent actors of destruction. However, instead of stabilizing a nation state through fictional scenarios (Boltanski, 2012/2014, p. xv), these novels perform critical tests of information states of simultaneity. Within this cryptographic reality, both writers play with suspicion against governmental actors taking part in fueling conspiracies. This cryptographic suspicion, which often unfolds in dark liminal spaces of whispered voices, underscores how gamic fiction immersed in digital economies conflates mystery with conspiracy to frame the “deep, hidden, threatening reality, which is unofficial but much more real” (Boltanski, 2012/2014, p. xv) as fragile and to be protected. In the early 2010 s, both Stephenson and Pynchon probe the set of beliefs associated with a novel capitalist order set within digital infrastructures. These networks employ a performative ouverte act, in which the deferral of ethical norms narrates mystery to consolidate their networks. Thus, these novels not only test the fragility of the system but also reconfigure fragility as strength, as explicated in greater detail in the following textual analysis of how each novel enacts such a test while diverting toward its respective aesthetic genre to subvert the test at key instances or, as Stephenson does, reestablish the test through the bestseller.

## Analysis I: The Disruptive Attack, *Reamde*, and the Picaresque Test

### The Virus as an Engine of Adaptability

In line with the kind of adaptability and flexibility Boltanski and Chiapello (1999/2007) diagnose for justificatory regimes, *Reamde* obscures how mystery framing digital money becomes a normative engine by which to absorb the destruction the attack causes. It establishes a test of justification and invites laughter at the fragility of the network while it emerges as a new powerhouse. The spirit of adaptability is most prominently forged when the misspelled virus turns into an author of the turbulence and turmoil and endangers gaming mogul Richard Forthrast’s virtual empire, creating a randomly emerging test of justification. The novel’s imaginary terrain becomes a projection of multiplying networks while parasitic actors consolidate power doing precisely what Boltanski observes in Serres’s theory: They mask the outside and inside of the network. First, Peter, Zula’s boyfriend, sells a database of stolen credit card numbers to a middleman (Stephenson, 2011, p. 73). However, the laptop used to verify them carries the Reamde ransomware, a virus/troll that encrypts the files and demands “the ransom […] be paid in the form of virtual gold pieces in T’Rain” (p. 97), trapping the data the Russian mob already purchased. The chance interception sets off the thriller. Then, when the chase for the origin of the virus starts crossing different actors with malicious intent, some of them changing sides, readers are suddenly thrown into a wild network of villains, trolls from Xiamen, and unlikely coincidences that redraw the novel’s imaginary terrain, spanning the entire globe. The hackers who created “Reamde” are entirely separate from the credit card transaction, yet the chance interception becomes the riddle that moves the plot, unbeknownst to most of the actors, but known to the reader. This is precisely the picaresque set-up Boltanski (2012/2014, p. 7) describes: “Stress is placed on the more or less chaotic nature of the various milieus in which the characters and, with them, all human beings are immersed, no matter when or where they live out their earthly lives. […] Life’s unfolding is thus comparable to a series of ‘throws’ – as in throws of dice – that is not governed by any principle of general causality but depend rather on whim or chance.” In this environment of coincidences, moral life is reduced to instantaneous choices. The hacker imaginary emerges as a rebel and hero, whose agency finds its fullest expression in the role of the troll.

While the line between narrated world and virtual terrain becomes fluid, so do the currencies earned in-game, as the narrative works “the boundary line between a real currency and a virtual currency,” which Stephenson calls “inherently interesting to me” (Adams & Kirtley, 2012; reflected in the novel, Stephenson, 2011, p. 739). With no clear inside or outside, the virus meant to extract the virtual money solidifies the existing game. T’Rain turns out to be as elliptical as its apostrophic writing and the virus ironically mirrors that, elevating the trickster imagery that Stephenson infuses into the plot. Hence, the plot of turbulence turns out to be a test in which the new spirit of digital capitalism symbolized by the ransomware attack gets the last laugh. The picaresque story establishes a test of chance, which substitutes the test of strength. Stephenson notes that there is “a certain picaresque quality in seeing some kid invent a piece of code that would touch millions of computers” (Klosowski, 2011). His remarks confirm how disruption is stylized as part of an attack that cannot simply be solved like a mystery; however, similar to a conspiracy, it sets up a second reality. The virus thus reorders the network. The MMORPG game eventually becomes an ultimate test of virtual space, within which digital assets are emotional vehicles of liberation, offering “creativity, reactivity, and flexibility,” while decentralization becomes a buzzword that denotes a new “explosion of bureaucracy” for technological self-governance (Boltanski & Chiapello, 1999/2007, p. 90). Eventually, T’Rain turns out to be a regime of justification for the promise of liberation at the expense of survivalist tactics in an ultimate global chase, which contracts time and space into a new pseudo-mythic reality. The novel, in its style, imitates the game while creating a new origin story that overwrites reality for a mysterious presence, in which magic money becomes a new form of gold, albeit serving the same neo-colonial objectives.

### T’Rain as a Regime of Justification and the Narrativization of New Origins

Stephenson’s heroes return to magical worlds, completing the “cycle of recuperation” (Boltanski & Chiapello, 1999/2007, p. 425), in which all communication becomes transactional. While framing their search for the “essence of the gift” (p. 426), they discover it as magic. Forthrast “investigates the history of money and particularly of gold” (Stephenson, 2011, p. 35) and constructs his own virtual republic, realizing that T’Rain “could only be as successful as the virtual currency. If people thought that the fruits of their questing—gold, magic swords, and so forth—might suddenly become worthless because of inflation or a security breach, they’d stop playing” (p. 35). In the “gold-famer-friendly” (p. 35) game of T’Rain, “questing for virtual gold and vicarious glory” (p. 30) is directed by a social engineering scheme. Crafting “ancient dead languages [to] [i]mbue the history of T’Rain with a grammar and a rhetoric” (p. 619) is delegated to the “Department of Narrative Dynamics” (p. 216) as the “fantasy-based aspect of the business” (p. 209) “Magic” is redefined as “a social construct invented by T’Rain’s sentient races to explain the different physics that governed the alien atoms” (p. 216). Forthrast’s gaming empire symbolizes a mystery economy that lures actors with the promise of empowerment. However, it reinforces predatory hierarchies and debt regimes: It “just extracted cash flow from players’ desire to own virtual goods that would confer status on their fictional characters as they ran around T’Rain acting out greater or lesser parts in a story” (p. 209). The game is not only built on “imaginary landforms” (p. 36) modeled after real geological data. This virtual planet “TERRAIN, the gigantic mess of computer code […] gave its name to T’Rain, the imaginary world where Corporation 9592 set up its new game” (p. 37). It also sets up a “feudal social structure” (p. 40). Here, the novel exposes the nexus of finance, storytelling, and games: Mystery regimes erase historical memory to install a pseudo-ancient terrain. The narrative department first adopts the role of an algorithmic machine of seemingly “neutral, dispassionate forces” that aim to “imbue history with grammar and rhetoric” (p. 617), but it has to admit failure when the assigned narrative of a fight between good and evil does not work.

The narrative control extends beyond the in-game lore. While linguistic archives and crypto-mining myths promise an exit from traditional labor structures, these liberatory promises sugarcoat deeper entanglements with precarious extraction, which as the novel shows is deeply intertwined with real-world destinies. However, as the origin stories prefigure rituals of distrust, the game itself becomes the trusted sphere, which has no trusted outside. Thus, the novel’s plot turns into a critical test in which mystery economies are exposed as participants in the conspiracies they produce. They construct origin stories to mask the extraction of time and labor, framed as opportunities in a gold rush, concealing the neo-colonial hierarchies on which they depend. This setup is similar to Stephenson’s *Snow Crash*, in which the virus “writes binary code and viral engineering back into history, making the reduction of conscious humans into automata the recapitulation of an ancient struggle. Computation has always been with us, the narrative implies, because computation is the basis for human neural functioning” (Hayles, 1999/2002, p. 274). Retrospectively, Stephenson moves that history into a gaming empire while presenting it critically as a debt regime, from which humans try to escape, only to dig themselves deeper into the imaginary terrain they colonize, exposing the revolution digital currencies promise as farce.

### The Revolution as Farce

*Reamde* exposes what later would be adopted by many cryptocurrencies: Trust is secured less by code than by narrative immersion with mythological quality carrying a spiritual truth. Stephenson describes a trend, later observed in play-to-earn blockchain games like *Axie Infinity*, in which “the heavy reliance on gaming labor as the source of value for digital assets result in a highly fragile rentiership system that is vulnerable to declining asset value” (Tan, 2025, p. 8). *Axie Infinity* would go on to destroy livelihoods in the Philippines, broader Asia, and the Global South, after a ransomware attack targeted the game in 2022, similar to the attack on Stephenson’s projects in 2011. Not unlike Stephenson’s fictional game, *Axie Infinity* marketed itself as “gold-famer” friendly. As in Stephenson’s fictional test, the real application also obscures exploitation chains that echo colonial resource extraction, profiting from the dream that the game might enable an escape from poverty. In reality, digital assets have an emotional aspect: While they are presented as atemporal magic, they extract time from players by “enacting techno-economic rentier capitalism” (Tan, 2025, p. 8). Play-to-earn games profit from social risks and the financial logic of tokenization (Lising, 2026), by “blurring gambling, gaming, and finance” (Zaucha & Agur, 2023, p. 1294) while relying on “the market’s ‘collective belief’ that serves as the self-referential foundation of value and enables the self-fulfilling performativity of the belief” (Lee, 2022, p. 114).

Stephenson’s critique is limited because the plot of *Reamde* reinforces “magical capitalism” (Lee, 2022). The novel itself maintains imaginaries, which portray the conquest of virtual and real territories as a heroic endeavor, such as denying “the validity of borders altogether” (Stephenson, 2011, p. 827) to rebuild land in a game-based image, while employing racialized stereotyped actors. Even though Forthrast’s niece, Zula, “an Eritrean orphan […] adopted by Richard’s sister” (Stephenson, 2011, p. 8) emerges as a heroine, there is no real correction of stereotypes at any level (see Liboriussen, 2017, pp. 146–149). Stephenson is only one example of how gamic fiction in the 2010s used a racialized image of the “Chinese hacker.” Despite hyping it up as a rebellious function, the novel describes their practice as “only the latest—but by far the most lucrative—moneymaking scheme they had launched from their rebel enclave” (Stephenson, 2011, p. 290). It explains: the “game was accessible to the all-important Chinese teenager market as it was to the podgy middle-aged Westerners who were dependent upon those Chinese teenagers for virtual gold,” (p. 40) supplying a “source of income” in a “happy arrangement” (p. 40) from Richard’s point of view. Stephenson’s critique of gamification, and the libertarianism Anna McFarlane (2016, p. 7) detects in *Reamde*, is limited as it simultaneously reinforces what Stark (2019, p. 163) calls a “proto-mythical framework.” The libertarian fantasy prevails, in which the narrativization of origins animates technological dynamics while excluding *queer* temporalities and Black futures (Keeling, 2019). This exclusion is established as the game reappropriates geological economies and imaginary landforms as its own ahistorical imaginary.

In Boltanski and Chiapello’s terms, *Reamde* reenacts a regime of displacement that justifies limited freedom characteristic of projective capitalism. Throughout their chase, all protagonists perform constant tests of displacement in which they circumvent orders to take advantage of unacknowledged strength (Boltanski & Chiapello, 1999/2007, pp. 107, 321). The precarious mobility demanded in the plot is resolved in a domestic union and stabilized through an alternate, ludic system. This domestic retreat serves to erase trauma in favor of an atemporal, lasting present while framing retrospectively the parasitic levelling and overturning of any “scale of justice” (Boltanski & Chiapello, 1999/2007, p. xiii) as necessary for a very small group of survivors. Although their domestic space appears to absorb the game’s narrative objective of going beyond good and evil (Stephenson, 2011, p. 617), in reality, the exit is recuperated within the logic it seeks to escape. The plot eventually levels the outside world to mirror T’Rain until both narrative threads (the chase for the ransomware attack) and the in-game worlds conflate in the end. Here, Richard fights his final battle under the avatar Egdod and wanders back to his origins: “both he and Egdod were wandering alone across their respective worlds […] Getting back in touch with the terrains from which they had sprung, autochthonously, early in their lives” (Stephenson, 2011, p. 1031). Retrospectively, the ransomware attack appears like a karmic game that defers morality for strength: “In the end, we all converge, and our ends are as much the same as our beginnings” (p. 924). Asked for their choice of a self-reliant “Christian right-wing thing” (p. 699), Richard’s brother, Jake, had recalled an Emerson-inspired moment of “Self-Reliance,” celebrating a community “not based just on geographical proximity or money, but on spiritual value.” The kitschy end rather oozes a whim of emphatic sympathy rather than establishing a critical frame of estrangement for the reader. This becomes most obvious in the reviewers’ fascination, noting “This novel has everything that a good hacker novel needs” (Howard, 2014) while writing “a new reality into existence,” inextricably linked with cryptocurrencies (Suderman, 2019).

While Stephenson’s travelogue recuperates disruption as a form of domestic retreat, *Bleeding Edge* performs a critical test of the inquiry itself, posing the question of what happens when critique and curiosity only reinforce the entanglements of conspiracy and mystery to create a market exchange of surveillance states and “behavioral modification” (Zuboff, 2019, p. 319). Stephenson’s novel tests digital capitalism’s self-justification, which is perhaps why Stephenson renounced his enthusiasm for cryptocurrency more than a decade later (Francis, 2023). Pynchon’s text unravels this test as a failure and measure of displacement. Adapting May’s cypherpunk question about the roadkill to cypherpunk dreams, one could say that *Bleeding Edge* portrays the casualty of the crypto-highway as another eulogy on a suspect in a mystery rejecting to giving up their “real story,” while “the dream, the promise—is destroyed” (Pynchon, 2013/2014, p. 432). *Bleeding Edge* begins with a mystery—the detective and fraud investigator Maxine Tarnow is set to look into the villain Gabriel Ice—but arrives at conspiracy as critique of power. In doing so, *Bleeding Edge* suggests that early cypherpunk dreams were likely co-opted into the very systems of surveillance and control they sought to evade. Pynchon thus helps to “reformulate the issues of liberation and authenticity, starting from the new form of oppression it unwittingly helps to make possible” (Boltanski & Chiapello, 1999/2007, p. 486). When one of Maxine’s hacker friends decides to go “dark and silent” (Pynchon, 2013/2014, p. 433) in the aftermath of the terrorist attacks in September 2001, Pynchon, more forcefully than Stephenson, asks for whom opacity is a choice and when it starts to become a condition of survival, pointing toward the failure of the inquiry that is beyond the imaginary of the cypherpunk. Games in *Bleeding Edge* become a vehicle to tacitly reflect on the *cost of connection* toward those who never appear, tacitly acknowledging that the projective city in DeepArcher remains a privileged refuge despite its pledge to take in anyone: “It is asylum, no matter, you can be poorest, no home” (Pynchon, 2013/2014, p. 373).

## Analysis II: *Bleeding Edge* as a Test of Deconstruction and the Privilege of Opacity

### The Code of Forgetting as a Failed Test of Justification

Pynchon exposes the failures of justification regimes by hailing mystery as an ultimate riddle, which secures a new form of opacity and protects from the past turning into a conspiracy. His paradoxical approach can hardly be co-opted by any movement, as the volatile history of cypherpunks illustrates. The novel’s focus on artist hacker dreams co-opted by capital is exemplified when Justin and Lucas, the two inventors of the digital utopia, DeepArcher, decide to go open-source (Pynchon, 2013/2014, p. 426) despite Ice’s offers (p. 79). Gabriel Ice, the “digital tycoon” (p. 136) in the “security business” (p. 137), embodies a “soulless source” (p. 114) and “mogul on the black-diamond slopes of the IT sector” (p. 311). His megalomanic ideas to build data centers in the Arctic (p. 310) lead to his proclamation that “the future is out there on the permafrost, a new geopolitical imperative—gain control of the supply of cold as a natural resource of incomputable worth […]” (p. 310). By contrast, DeepArcher preserves a privileged nostalgia of creating an (a)historical space of resistance against Ice’s schemes, offering a hacker’s retreat—a “sanctuary” (p. 74) and “refuge” (p. 373) from capitalist extraction. It employs a grotesque, surreal imaginary when it ironically suggests that within this space the dead and living can meet through avatars. Pynchon refers to debates on the creation of digital afterlives as portrayed in *True Names* (Vinge, 1981): the encrypted uploading of minds into cyberspace, prefiguring cypherpunk’s dreams of living on after death through encrypted pseudonymity in another plane and in crypto-anarchy. Pynchon’s (2013/2014, pp. 108, 376) idyllic hacker retreat offers an opportunity to escape from time-credits and debt—“living on borrowed time”—into a space that knows no borrowing or owning. Anyone in DeepArcher finds themselves as surreal space travelers: “Down there, we cannot be gamers, we must be travelers” (p. 373), offering the grid for a future, a “virtual hometown … still safe from the spiders and bots that one day too soon will be coming for it, to claim-jump it in the name of the indexed world” (p. 476).

### A Projective City as a Privileged Refuge

DeepArcher adopts an aesthetic function, “where the tests that matter involve constructing or loosening connections in a network world, obviously does not mean that the establishment of networks represents a radical new departure,” as Boltanski and Chiapello (1999/2007, p. 107f) show in their discussion of the *projective city*. Pynchon problematizes Boltanski and Chiapello’s (1999/2007, p. 152) observations that “a suitable allocation of free time also concerns access to information and money,” noting that saving time must be “constantly reinvested.” This is enhanced in an emerging digital economy where time is constantly reinvested in digital assets played, leading into what will then become rentier capitalism, borrowing time and assets. Maxine Tarnow’s investigation represents a critical test of this allocation while uncovering a gap between the regime’s justification, which proclaims the freedom of data centers, and its reality, requiring either the colonization of energy or a flight into predation and amnesia.

The roadmap of DeepArcher, built on the storylines of “*Akira*, *Ghost in the Shell* Metal Gear Solid” (Pynchon, 2013/2014, p. 69), structures a new temporal experience grounded in the premise of forgetting. Using a remailer technology to create “an invisible self-recoding pathway, no chance of retracing it” (p. 79), the “History-free” (p. 373), “timeless refuge” (p. 373) is essentially about an exchange of life in debt for death, travelling in atemporality. When time and meaning collapse into “late-capitalist contradiction” (p. 395), Pynchon’s artistic critique becomes an intervention into the conspiracies with which the novel plays. One may recall Shaoul’s (2003) response on the cypherpunk list to the potential data leak of his purported address: “Pynchon may not be as stupid as that. Has anyone gone to check up on Mr. Vineland lately?” The reference to *Vineland*’s (1990) ironic take on undercover identities and the co-option of countercultural movements cautions against trusting the data per se. *Bleeding Edge* progresses from “Mr. Vineland” as the narrative departs game time of a “falsely deathless perimeter” (Pynchon, 1990, p. 293) for a new signal that includes death as another form of life.

All of Pynchon’s detectives, and Maxine is only one of them, never truly uncover a crime and therefore stabilize a past event. Rather, the narrative helps to create a poetics of the other world. The ghosts of the past become the ghost of the present to gesture toward how *randomness* reconfigures the game or “various degrees of uncertainty haunting situations in social life” (Boltanski & Chiapello, 1999/2007, p. 30). *Bleeding Edge* hints at a justificatory opening for surveillance regimes to reconfigure temporal perception when Horst, Maxine’s ex-husband, detects statistically abnormal put options before 9/11 (Pynchon, 2013/2014, p. 315), deviating from normal market randomness. The anomalies invoke what Boltanski (2014, pp. 223, 257) theorizes as a grammar of social causality: the attribution of market effects to shadowy collective actors and “fuzzy circles of influence” that escape legal recognition but shape historic outcomes.

In contrast to markets structuring a new “community of time” in screen realities (Knorr-Cetina & Bruegger, 2002, p. 929), the projective city becomes an inquiry into their theatricality of time while reversely imagining a techno-romantic, transcendental sphere. The code of forgetting, the redaction of all traces, becomes an intentional failure of justification, creating instead a critical space beyond the speculative finance Ice represents. The “looming and prophetic landfill, that perfect negative of the city in its seething foul incoherence, and find a set of invisible links to click on and be crossfaded at last to unexpected refuge” (Pynchon, 2013/2014, p. 167) emerges as a temporary, Alhambra-like “oasis” (p. 406) for dreams that could have been but never will. It is well aware of its feeble state, as one friend of Maxine’s hacker friends decides to go dark and wonders “maybe what we’ve been living through is just a privileged little window” (p. 432). Against the traumatic historic events, *Bleeding Edge* looks back on how the vision of cypherpunks to reverse time’s arrow through cryptography transformed into a failure (see also Brunton, 2019, p. 204).

Pynchon’s gloomy outlook might be summarized in the meme-glitch of irreversibility that he introduces into the plot as the arcade shooter game *Zero Wings*. The reference to the meme “All Your Base Are Belong to Us” (Pynchon, 2013/2014, p. 384) comes to represent Ice’s massive embezzlement (p. 385). The meme, now only a print on Avi’s T-shirt, encodes a warning toward all-pervasive destruction in terms of memories, which does not ask whose history is erased. At the same time, it contrasts with the novel’s multiple temporalities, while arcade games, conspiracies, and references form a resilient network that creates its own opaque, riddle-like space.

The new technological infrastructure adopts a mystical reality in its own right, which Pynchon integrates into his aesthetic critique, while setting it against the acknowledgment of privilege (Pynchon, 2013/2014, p. 39). At the very end, all protagonists discover that their endeavor has left nothing but a “capitalist party mask” in a “denial of the passage of time” (p. 311), generating figures of loneliness and debt (p. 474). Ice also has to acknowledge that he too is tracked by his “overlords” (p. 474), demonstrating that “a continuously trackable life is a dispossessed life, no matter how one looks at it” (Couldry & Mejias, 2019a, p. 345). While DeepArcher itself leaves the material *costs of connection* (Couldry & Mejias, 2019b) largely unaddressed in explicit ways, those costs appear in fragments—for example, in the story of agent Windust’s operations close to the Mexican border (Pynchon, 2013/2014, p. 444), stirring geopolitical violence as the other side of a digital empire’s infrastructure of data fraud and collection.

### Ethics of Opacity and the Future of the Archer as a Synthesis of Artistic Dissonance

Progressing from game time, *Bleeding Edge* presents artistic dissonance between capitalist technoscience and a surreal experience of embodiment, which proposes the glitch as a new form of opacity and projective future. Accordingly, DeepArcher conceals a glitch between a reality that needs to obscure itself and a reality driven by “alternative histories” (Pynchon, 2013/2014, p. 243). Described in dialectical terms, it is not a game, “though it does have forerunners in the gaming area” (p. 69) or a map of “no map” (p. 77). DeepArcher points to the need to combine aesthetic and social critiques without giving one “exclusive expression” over the other (Boltanski & Chiapello, 1999/2007, p. 536). Pynchon’s (2013/2014, p. 357) digital “flow of starscape” problematizes the contrast between what Couldry and Mejias (2019b, p. 143) point out as “the behavioristic reduction of human experience to what can be tracked [and] serves the capitalization of life perfectly,” and the artistic rush of exploration of an abyss, “a horizon between coded and codeless” (Pynchon, 2013/2014, p. 357). The digital proposal simultaneously points toward minimal integrity of the self to be preserved through mystery as a means of holding onto time by releasing it. Pynchon extends the call of sociologists to create systems “unfettered by capitalist dynamics of destruction” (Boltanski & Chiapello, 1999/2007, p. 531) by suggesting that within the destructive dynamics lies an aesthetic gem to refract the present toward greater hope. The novel proposes the embrace of an artistic reality of dissonant voices to hedge against the zero-sum games of a capitalist system.

*Bleeding Edge* points to the issue of who remains in a position to inquire about reality and who remains unseen. This right remains urgent—not only outside a Western imaginary but also within traumatic events such as the many faceless victims of 9/11 Maxine imagines seeing during her trips through the city, like a “return of the dead” (Pynchon, 2013/2014, p. 339). If Pynchon asks who remains unseen, then the novel simultaneously performs its limits on the margins of a history that cannot be surrendered. Glissant’s (1990/1997, p. 190) concept of the “right to opacity that is not enclosure within an impenetrable autarchy but subsistence within an irreducible singularity” extends that concern. These limitations come into focus through what Hartman (2008, p. 13) calls the “afterlife of property,” acknowledging the impossibility of escaping history when your very body is the archive of trauma, trapped in the notion of “propertied person” (Hartman, 1997, p. 129). The novel asks whether death emerges as an ultimate opaque frontier toward forgetfulness or whether bodies might be archived in the mute mystery suspect of the city itself. Pynchon (2013/2014, p. 75) portrays a trans-national “departure” (DeepArcher) leading to “darkness pulsing,” which morphs opacity into different temporalities. This may limit his vision to the luxury of aesthetic retreat as glitched realities offering the release of time into space. Pynchon tests this trans-national space of the ungovernable and anarchic, thereby navigating toward a trajectory extended by Keeling’s (2019, p. 32) call to “queer temporalities.” While not grounded in a queer perspective, Pynchon’s vision invites a discourse on Glissant’s (1990/1997, p. 194) claim that “death is the outcome of the opacities.” Keeling (2019, p. 36) frames these temporal interventions as “blossoming in spite of what presently feels to be the future” anchoring “Black existence” on “opacity that invites and frustrates knowledge, transparency and measure.” While neither *Reamde* nor *Bleeding Edge* imagines Black futures, both point to the limitations of their visions. *Bleeding Edge* demonstrates how against Ice’s security business, opacity becomes a survival technique. So does the inquiry or critical test that Maxine performs. Pynchon’s latest novel, *Shadow Ticket* (2025), shows how these tests iterate in pre-digital monopolies, with crime syndicates enacting the same mystery economies of opaque control, dismantling the tactics of justificatory regimes essentially only adopting new techniques. The novel once more blends mystery with conspiracy to preserve a memory, which in speculative finance of digital economies risks erasure as a mere asset in a game.

## Conclusion: Fiction as Critical Test and Temporal Archive

Both *Reamde* and *Bleeding Edge* reveal how tests of justification enable color-blind and neo-colonial visions of the future of cyberspace, in which capitalism is foremost sustained through emotional intensity. Despite differences in style and genre, both novels offer a prism to diffract the narratives of mystery, conspiracy, and the implicit (a)temporalities that digital assets generate. Stephenson and Pynchon perform critical tests of digital economies, probing whether their double-edged allure stems from a self-proclaimed financial revolution, the promise of empowerment and decentralization, or the thrilling unpredictability of the financial game itself. Magic money becomes a stage actor in a theater of time, narrativizing origins and rewriting a new history into gamic worlds that aim to stabilize an incoherent and increasingly contingent reality. As such, both authors interrogate narrated infrastructures for future money that might fundamentally reconfigure the social communication structure and lead to an emotional turn in digital finance. In *Reamde*, the hacker’s disruption, framed as picaresque rebellion, is ultimately resolved in a domestic retreat of religious and transcendentalist visions, recuperating the critique in the very logic the novel’s thriller seeks to escape. Stephenson’s imaginary landform participates in the erasure of indigenous terrains by establishing a mythical, digital present, which hides neo-colonial power networks in gaming environments. Pynchon, by contrast, shows that when all structures break down, we are left not with a network but with the simulation of travelling into a counter-world. His utopia, DeepArcher, displaces games for artistic dissonance of the afterlife. His digital refuge aligns with Boltanski’s projective city and hints at the problem of opacity as a means of survival. The hacker utopia carries the hope of encrypted privacy as a potential test of the failure of justification, subverting it through a new temporal proposition of instantaneous release from all coded connections. It also raises an important question: For whom may such opacity be a choice? In establishing different critical tests, both writers exemplify how fiction productively interacts with sociological approaches to reveal the recursive performativity of digital money. They demonstrate how digital assets profit from mystery economies generating destructive cycles (crashes, scams, and resets), while masking neo-colonial extraction. They point to how gamic worlds tend to erase the histories of those they displace by substituting new realities, narrativized origins for spaces to reinvest in time in disregard of the costly imperatives gaming moguls or predatory financiers claim for themselves. As real-world examples have since multiplied—exchanges defaulting due to fraud and blockchain games experiencing ransomware attack—these novels stand as early warnings. They reveal blockchain technologies for what they are: tools in future cultural economies, building their own time stamps and histories into malleable times of internet histories and receding archives. Thus, contemporary fiction on speculative finance and the ethics of digital economies uses narrative to prove that while “money is an imperfect form of memory” (Kocherlakota, 1998, p. 233), mystery economies, contrary to blockchain’s promise of permanence and indelible records, are prone to systematic erasure of memories, making it even more urgent to develop a cultural archive to access its flawed histories. By preserving narrativity against atemporal erasure, literary critique regains agency through an aesthetics of references that remembers dark histories, ensuring that humanities remain essential in exposing the tempered, contested reality we all inhabit. In restaging theaters of time for magic money in counterfeit worlds of mystery and conspiracy, fiction offers a critical test that is untraceable, fragile, and contrafactual. Hence, it is perhaps most trustworthy in telling the *real* story of future money as an opaque critique of our present.

## Data Availability

No datasets were generated or analysed during the current study.
